# Multiplexed Single-Cell *in situ* RNA Profiling

**DOI:** 10.3389/fmolb.2021.775410

**Published:** 2021-11-11

**Authors:** Yu-Sheng Wang, Jia Guo

**Affiliations:** Biodesign Institute and School of Molecular Sciences, Arizona State University, Tempe, AZ, United States

**Keywords:** fluorescence *in situ* hybridization, fish, transcripts, transcriptomics, genomics

## Abstract

The ability to quantify a large number of varied transcripts in single cells in their native spatial context is crucial to accelerate our understanding of health and disease. Bulk cell RNA analysis masks the heterogeneity in the cell population, while the conventional RNA imaging approaches suffer from low multiplexing capacity. Recent advances in multiplexed fluorescence *in situ* hybridization (FISH) methods enable comprehensive RNA profiling in individual cells *in situ*. These technologies will have wide applications in many biological and biomedical fields, including cell type classification, signaling network analysis, tissue architecture, disease diagnosis and patient stratification, etc. In this minireview, we will present the recent technological advances of multiplexed single-cell *in situ* RNA profiling assays, discuss their advantages and limitations, describe their biological applications, highlight the current challenges, and propose potential solutions.

## Introduction

It has long been recognized that biological systems ranging from genetically identical yeast and bacteria cells to multicellular organisms are composed of molecularly and functionally different cells (Altschuler and Wu, 2010). Such cell heterogeneity plays many important roles in various biological processes, such as immune response (Ma et al., 2011), cancer metastasis (Valastyan and Weinberg, 2011), drug response (Cohen et al., 2008; Sharma et al., 2010; Shaffer et al., 2017), and stem cell differentiation (Chang et al., 2008), among others. Every individual cell in these biological systems has a huge collection of distinct biomolecules, which are regulated by a large number of varied signaling pathways. Due to such complex signaling network and cell heterogeneity, single-cell transcriptomics technologies are in critical need to advance our understanding of health and disease.

The specific locations of cells in a tissue and biomolecules within a cell are critical for effective cell-to-cell interactions and intracellular signaling networks. These inter and intra-cellular interactions can determine the regulation, organization and function of the biological systems (Mondal et al., 2018a). For instance, the gene expression in individual cells along the embryonic body axes are tightly regulated, so that the generated gene expression gradients can direct the formation of specific organs (Reeves et al., 2012). Another example is that neurons develop and maintain their polarized cellular structures by precisely regulating the expression and transportation of RNA and proteins at their varied compartments. In this way, the effective signal transmission between presynaptic and postsynaptic neurons can also be formed in different neural circuits. Therefore, to accelerate our understanding of the composition, architecture and interactions in the complex biological systems, multiplexed single-cell RNA profiling at their native spatial contexts is critically needed.

To enable single-cell spatial transcriptome analysis, a number of methods, such as laser capture microdissection (LCM), *in situ* sequencing, and *in situ* capturing technologies, have been explored (Asp et al., 2020; Liao et al., 2021). Although these approaches dramatically advance our ability to investigate gene expression in its native spatial contexts, some nonideal factors still exist. For example, as LCM requires individual cells of interest to be cut out from tissues (Emmert-Buck et al., 1996; Bonner et al., 1997; Chen et al., 2017), the sample throughput of this approach is low. To sequence the transcripts in their original cellular locations or captured on DNA-barcoded chips, the RNA sequences have to be reverse transcribed and amplified first (Lee et al., 2014; Stahl et al., 2016; Wang et al., 2018; Rodriques et al., 2019; Vickovic et al., 2019; Liu et al., 2020). Due to the limited RNA capturing, reverse transcription or signal amplification efficiency, the detection sensitivity of *in situ* sequencing and *in situ* capturing is relatively low.

In this minireview, we will describe the hybridization-based spatial transcriptomics technologies, which enables thousands of RNA species to be profiled in single cells at the single-molecule sensitivity with the optical resolution. Readers are referred to other excellent reports (Asp et al., 2020; Liao et al., 2021) concerning alternative *in situ* transcriptomics methods, such as LCM, *in situ* sequencing, and *in situ* capturing, etc. Here, we will introduce the design, advantages and limitations of the hybridization-based spatial transcriptomics approaches. Their applications to study cell signaling pathways, cell heterogeneity and cell-to-cell interactions will also be highlighted, together with their broad impact on understanding, diagnosis and treatment of different diseases. Finally, we will discuss the current challenges of these methods and propose potential solutions.

## *In Situ* Hybridization Technologies

**FIGURE 1:**
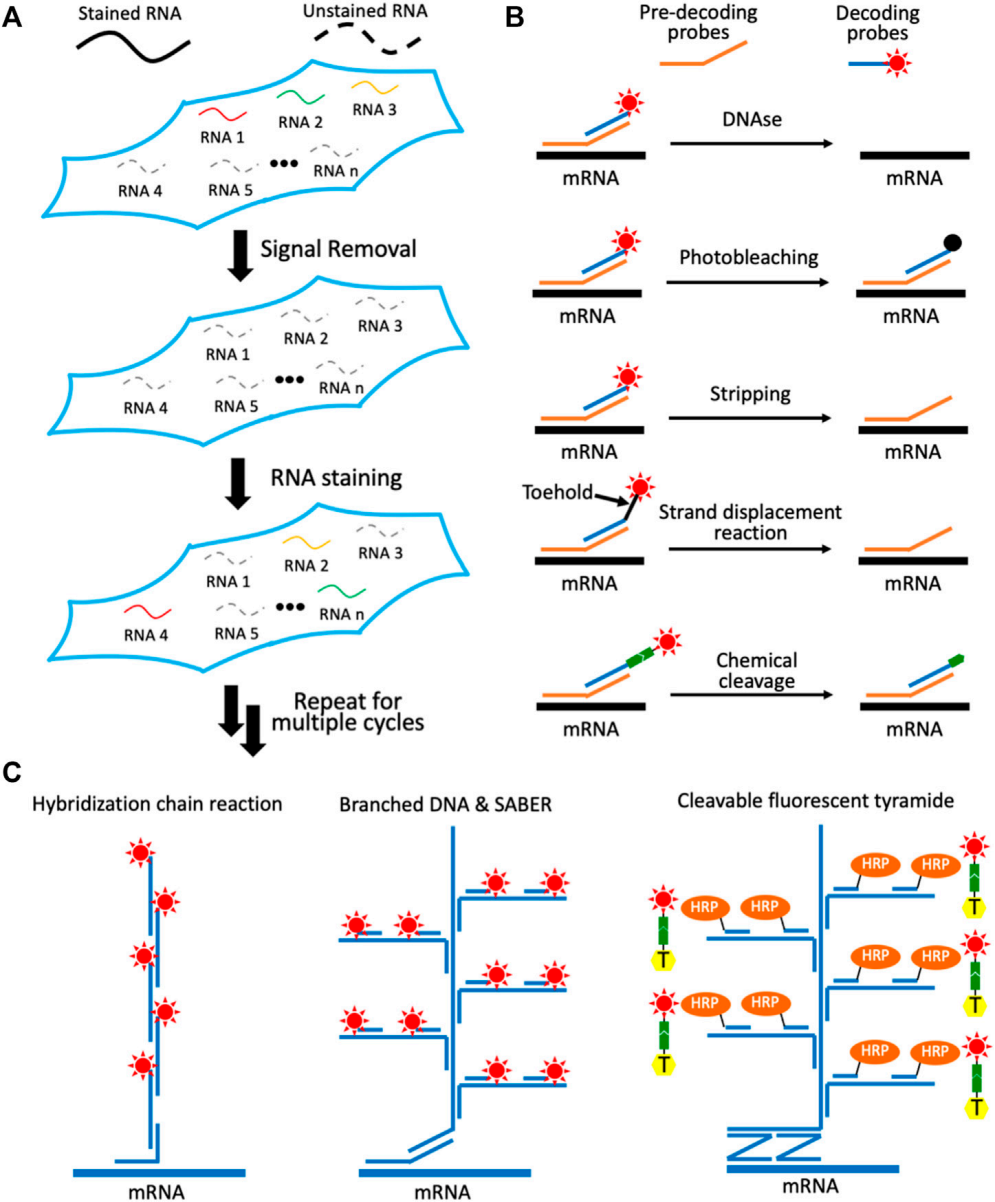
Multiplexed single-cell *in situ* RNA profiling. **(A)** Spatial transcriptomics analysis is achieved by continuous RNA staining. **(B)** Different approaches have been explored to remove the fluorescence signals in each RNA staining cycle. **(C)** Varied signal amplification methods have been developed to improve the detection sensitivity. With multiple transcripts stained in each cycle by probes with different sequences and through reiterative cycles, a large number of varied RNA species can be sensitively detected *in situ*.

To visualize a large number of varied RNA species directly in their original cellular environment, a number of *in situ* hybridization technologies have been developed. These methods include error-robust fluorescence *in situ* hybridization (MER-FISH) (Chen et al., 2015), sequential hybridization (Eng et al., 2019) and reiterative hybridization (Xiao and Guo, 2015; Shaffer et al., 2017; Mondal et al., 2018b; Xiao and Guo, 2018; Xiao et al., 2020). Each cycle of these approaches is mainly composed of three steps (Figure 1A). First, varied RNA species are stained using fluorescent oligonucleotides by *in situ* hybridization. Second, fluorescence images are captured in every fluorescence channel, so that each RNA molecule is visualized as a fluorescent spot. With multiple fluorescent oligonucleotides hybridized to the different regions on each transcript, the generated staining signals are significantly stronger than the nonspecific background, and thus can be easily identified by computational algorithms. Finally, the staining signals are removed to initiate the next cycle. Through continuous cycles of target hybridization, fluorescence imaging and signal removal, comprehensive RNA *in situ* profiling can be achieved. For instance, when distinct RNA species are stained in continuous analysis cycles, a total of A × B transcripts can be profiled in a sample, where A is the number of different fluorophores applied in each cycle and B is the number of analysis cycles. And by staining the same set of RNA species in every hybridization cycle, each transcript with its fixed cellular location can be identified as a fluorescent spot with a unique color barcode. In this case, the multiplexing capacity of the assay increases exponentially with the cycle numbers. With A fluorophores used in every cycle and B analysis cycles, an overall A^B^ varied RNA species can be quantified in individual cells in their native spatial contexts. Using these methods, it has been demonstrated that thousands of different RNA species are directly visualized in single cells (Eng et al., 2019).

## Signal Removal Methods

One of the critical requirements for the success of these spatial transcriptomics technologies is to efficiently erase the staining signals at the end of each cycle. In this way, the minimum signal leftover will not result in false positive signals in the following cycles. Another requirement of these approaches is that the RNA integrity has to be maintained during the signal removal process. Consequently, the varied RNA targets can be successfully stained in continuous analysis cycles. To fulfill these two requirements, a number of methods have been explored (Figure 1B). For example, DNase is applied to degrade the fluorescent oligonucleotide probes to erase the staining signals (Lubeck et al., 2014). Due to the high efficiency and specificity of the DNase, the fluorescence signals can be effectively removed without damaging the RNA integrity. However, in most of the hybridization based spatial transcriptomics approaches, a large library of pre-decoding probes is first hybridized to all the RNAs of interest. Subsequently, these pre-decoding probes will recruit the fluorescently labeled decoding probes to stain the targets (Figure 1B). When DNase is applied, both pre-decoding probes and decoding probes are degraded. As a result, at the beginning of each cycle, the pre-decoding probes have to applied again, which is time-consuming and costly.

To keep the pre-decoding probes hybridized to their targets during signal removal, photobleaching has been explored to erase the staining signals (Figure 1B) (Xiao and Guo, 2015; Xiao et al., 2020). This approach enables the efficient signal elimination while maintaining the RNA integrity. And as the pre-decoding probes are not damaged or removed during the analysis cycles, they only need to be applied in the first cycle for the whole assay. Nonetheless, this approach requires the tissue sections to be photobleached area-by-area and the fluorophores to be bleached one-by-one, which leads to the long assay time and limited sample throughput. To tackle these issues, probe stripping with hot formamide has been developed (Shaffer et al., 2017; Eng et al., 2019). In this approach, pre-decoding probes are hybridized to the targets and decoding probes with varied melting-temperatures. As a result, the formamide solution at the desired temperature and concentration can only remove the decoding probes and leave the pre-decoding probes hybridized to their RNA targets. However, with repeated probe stripping in different cycles, a certain percentage of the pre-decoding probes may also dehybridize from the transcripts, especially in the later cycles. These lost pre-decoding probes will lead to false negative signals and decrease the analysis accuracy.

To remove decoding probes more specifically, strand displacement reactions have been applied (Xiao and Guo, 2018). In this approach, the decoding probes are released by perfectly complementary oligonucleotide erasers. After target staining, the toehold regions at the 3′ or 5′ end of the decoding probes are designed to remain unhybridized (Figure 1B). The oligonucleotide erasers will hybridize to the single-stranded toehold regions on the decoding probes, branch migrate and finally dehybridize the decoding probes from pre-decoding probes. As the probe removal process is sequence-specific, this approach allows the efficient removal of the decoding probes, while almost all of the pre-decoding probes remain hybridized to the RNA targets. Nevertheless, the application of this method to study thick tissues is hindered, due to the slow diffusion of bulky oligonucleotide erasers and the released decoding probes. To address this issue, chemical cleavage methods have been developed (Moffitt et al., 2016; Mondal et al., 2018b). In these methods, a mild chemical reaction is applied to chemically release the fluorophores tethered to the staining probes through a cleavable linker. As the cleavage reagent is a small molecule and only the fluorophores are removed instead of the whole fluorescent oligonucleotides, this approach allows the rapid spatial transcriptomics analysis in 3-D tissues.

## Signal Amplification Approaches

Using multiplexed FISH without signal amplification, it can be challenging to detect RNA in highly autofluorescent tissues, especially for the short transcripts with less possible probe-binding sites. Additionally, signal amplification can also reduce the imaging time and enhance the sample throughput. To enable highly sensitive spatial transcriptomics analysis, several approaches have been explored (Figure 1C). For instance, hybridization chain reaction (HCR) is applied to assemble a pair of fluorescent oligonucleotides into long concatemeric chains, to achieve enzyme free signal amplification (Shah et al., 2016). For branched DNA amplification, the amplifier oligonucleotides with multiple probe binding sites are assembled on the target RNA. Subsequently, the generated branched DNA complex will recruit many copies of fluorescent oligonucleotides to stain the transcript. The amplifier oligonucleotides can be prepared *ex vivo* and used in the hybridization reactions directly (Xia et al., 2019; Xiao et al., 2020), or they can also be synthesized *in situ* using primer-exchange reaction (PER) (Kishi et al., 2019). With these signal amplification approaches, the FISH staining intensities can be improved by up to two orders of magnitude.

Formalin-fixed paraffin-embedded (FFPE) tissues are the most common archived specimens in clinical tissue banks (Blow, 2007). Due to their extremely high autofluorescence and partially degraded RNA, FFPE tissues have not been successfully profiled using the methods described above. To enable multiplexed *in situ* RNA profiling in FFPE tissues, branched DNA complex and the horseradish peroxidase (HRP) are combined to amplify the signal (Xiao et al., 2021). To ensure the staining specificity, this approach uses pairs of oligonucleotide probes to recognize the target (Figure 1C). Only when these two probes in a pair hybridize to the transcript in tandem, the branched DNA can be assembled. By enzymatic deposition of cleavable fluorescent tyramide using HRP, the sensitivity of this approach is enhanced by another 1-2 order of magnitude, compared to the signal amplification methods discussed above. Through cycles of target staining, fluorescence imaging, fluorophore chemical cleavage and probe stripping, highly multiplexed *in situ* RNA analysis in FFPE tissues has been achieved.

## Biological Applications

**FIGURE 2:**
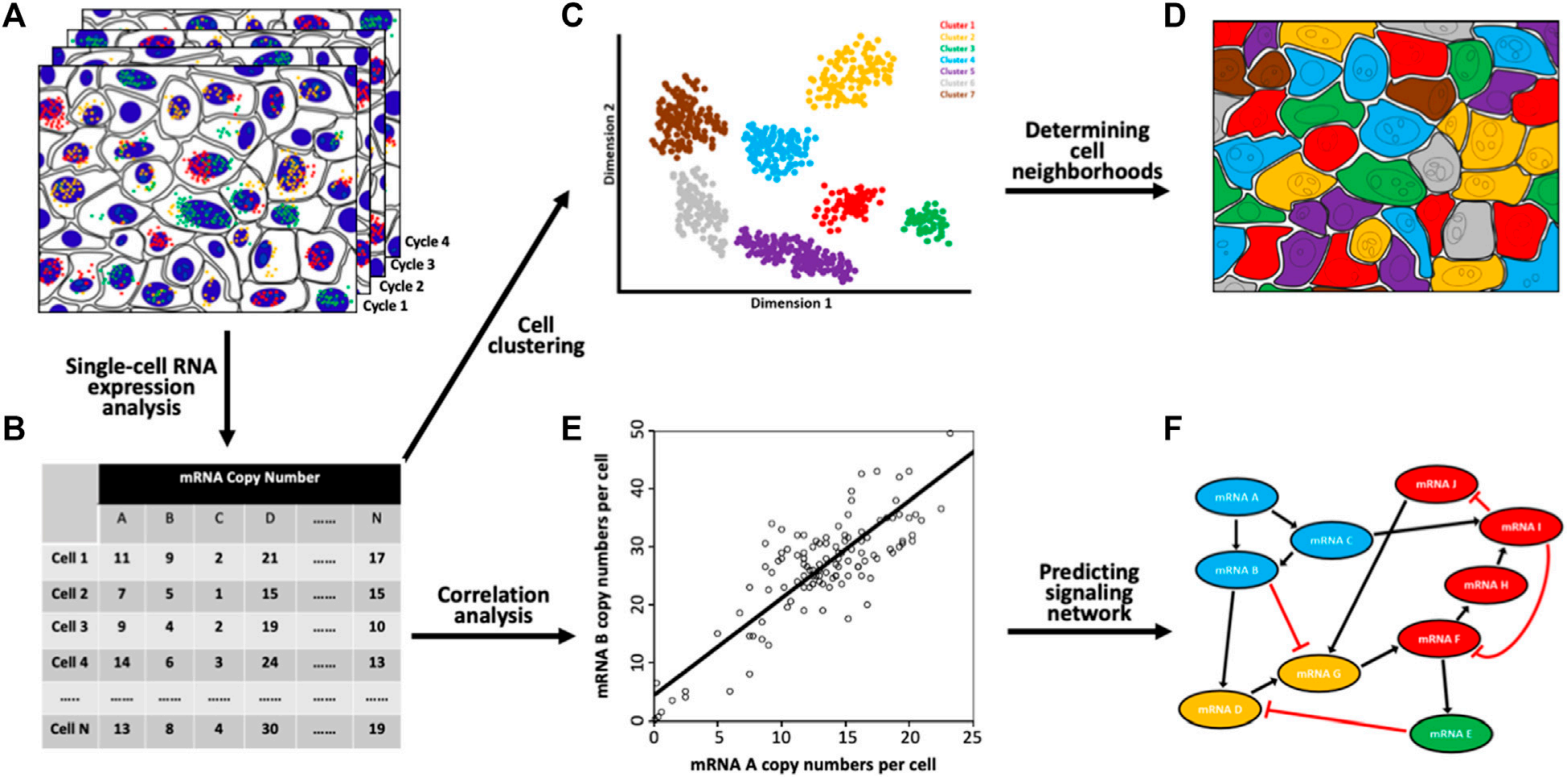
Biological applications of the spatial transcriptomics technologies. **(A)** Through continuous RNA staining and imaging **(B)** comprehensive RNA profiling has been achieved in single cells *in situ*. **(C)** Based on their unique RNA expression patterns, the single cells in a population are partitioned into various subgroups. **(D)** By mapping the individual cells back to their original tissue locations, distinct cell neighborhoods composed of cells from specific subgroups are identified. **(E)** Pairwise RNA copy numbers correlation analysis is performed with every spot representing one cell and its RNA copy numbers shown in the *x* and *y* axes. **(F)** With the generated correlation coefficients, a signaling network is established.

The multiplexed single-cell *in situ* RNA profiling technologies are powerful tools to investigate the distinct cell types and subtypes in complex biological systems. Through continuous RNA staining cycles (Figure 2A), a large number of varied RNA species can be profiled in individual cells in a sample (Figure 2B). Based on their unique RNA expression patterns, the individual cells can be classified into different clusters (Figure 2C) (Amir et al., 2013). Using this approach, the cell heterogeneity has been explored in the mouse hypothalamic preoptic region (Moffitt et al., 2018), retina (Kishi et al., 2019), cortex, subventricular zone, olfactory bulb (Eng et al., 2019), and spinal cord (Xiao et al., 2021). Additionally, by mapping the identified cell types back to their original tissue locations (Figure 2D), the varied cell neighborhoods composed of specific cell types can be defined. Such results will bring new insights into tissue architecture and cell-to-cell interactions.

Another exciting application of the spatial transcriptomics technologies is to study signaling pathways. To explore gene expression covariation with populations of cells, external stimuli, such as interfering RNA, small molecule inhibitors or knockout models, are required to introduce gene expression variation. With natural stochastic gene expression in individual cells (Elowitz et al., 2002; Blake et al., 2003; Becskei et al., 2005), pairwise RNA copy number correlation analysis can be performed in single cells (Figure 2E). Such analysis can be applied to interrogate multiple pathways without stimulating each of them separately (Figure 2F). This approach can also suggest new signaling networks, constrain regulatory pathways, predict the functions of unknown genes, and study the molecular mechanisms of drug resistance (Chen et al., 2015; Xiao and Guo, 2015; Shaffer et al., 2017).

## Conclusions and Future Perspective

The spatial transcriptomics technologies discussed above have dramatically enhanced our ability to understand the composition, regulation, architecture and interaction in complex biological systems. However, some aspects of these approaches need to be further improved. For example, the current spatial transcriptomics methods do not allow *de novo* analysis and have no base resolution. To partially address these issues, single-cell sequencing can be carried out first to identify the panel of RNA targets and the transcript variants. Subsequently, the specific probes for these targets and transcript variants can be designed and applied in the multiplexed RNA imaging assays. Another challenge for the single-cell *in situ* transcriptomics technologies involve image analysis. To determine the cellular boundaries in the tissues, the current approaches use the stained nucleus to indicate the presence of individual cells. And computational algorithms based on the distance from the nucleus are applied to estimate the cellular boundaries. However, in a typical 10 μm tissue, some cells may not have their nucleus included in the tissue. Also, those algorithms could be less accurate when analyzing cells with highly polarized structures, such as neurons. To mitigate these issues, some membrane proteins and cytoskeleton proteins can be co-stained with the transcripts, to facilitate the precise cell segmentation process.

With the recent technological advances, spatial transcriptomics technologies promise to provide many important insights into biology and medicine. By revealing the signaling networks, cell type compositions, spatial organization and cell-cell interactions in complex organisms, including brain tissues, solid tumors and developing embryos, we can dramatically accelerate our understanding of normal physiology and disease pathogenesis. By pinpointing the altered RNA expression or their cellular locations in patient samples, novel biomarkers can be identified for precise diagnosis and patient stratification. Additionally, new drug targets could be discovered for more effective targeted therapy. The spatial transcriptomics technologies described here can be also integrated with other molecular imaging methods, such as multiplexed protein imaging approaches (Mondal et al., 2018a; Pham et al., 2021), to enable the single-cell *in situ* comprehensive molecular profiling in the same specimen. We envision that the spatial transcriptomics technologies will broadly complement other omics approaches, and will have a wide range of applications in biomedical research and precision medicine.
